# How Molecular Motors Work in the Crowded Environment of Living Cells: Coexistence and Efficiency of Normal and Anomalous Transport

**DOI:** 10.1371/journal.pone.0091700

**Published:** 2014-03-13

**Authors:** Igor Goychuk, Vasyl O. Kharchenko, Ralf Metzler

**Affiliations:** 1 Institute for Physics and Astronomy, University of Potsdam, Potsdam-Golm, Germany; 2 Institute of Applied Physics, National Academy of Sciences of Ukraine, Sumy, Ukraine; 3 Department of Physics, Tampere University of Technology, Tampere, Finland; Weizmann Institute of Science, Israel

## Abstract

Recent experiments reveal both passive subdiffusion of various nanoparticles and anomalous active transport of such particles by molecular motors in the molecularly crowded environment of living biological cells. Passive and active microrheology reveals that the origin of this anomalous dynamics is due to the viscoelasticity of the intracellular fluid. How do molecular motors perform in such a highly viscous, dissipative environment? Can we explain the observed co-existence of the anomalous transport of relatively large particles of 100 to 500 nm in size by kinesin motors with the normal transport of smaller particles by the same molecular motors? What is the efficiency of molecular motors in the anomalous transport regime? Here we answer these seemingly conflicting questions and consistently explain experimental findings in a generalization of the well-known continuous diffusion model for molecular motors with two conformational states in which viscoelastic effects are included.

## Introduction

After the publication of Albert Einstein’s theory of Brownian motion in 1905 [1], Jean Perrin reported the first systematic microscopic studies of individual diffusing particles in 1908 [2]. Today, modern single particle tracking techniques routinely reveal insight into the stochastic motion of submicron tracers in aqueous solutions at unprecedented resolution, thus allowing one to directly observe the transition from initial ballistic to diffusive Brownian motion [3] and to resolve the effects of hydrodynamic backflow [4]. Measuring the passive and driven motion of microprobes has become a standard means to characterize soft matter [5]. Particular attention is currently paid to the relaxation and diffusion dynamics in dense colloidal systems [6], [7] and inside living biological cells [8], [9].

The intracellular fluid (cytosol) of biological cells is a superdense [10] aqueous solution containing biomacromolecules such as proteins and RNA at volume fractions of up to 40%, a state often referred to as macromolecular crowding [11], [12]. Indeed, the state of crowding in the cytosol effects severe changes of the diffusion behavior of submicron particles [8], [13], [14]. Thus, anomalous diffusion of the form 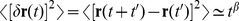 with 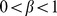 is observed in living cells for the passive motion of single biopolymers, endogenous granules, viruses, and artificial tracer particles [15]–[21]. Compared to normal Brownian motion with 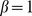, these particles therefore subdiffuse [22]. Such anomalous dynamics presents a challenge to the development of controlled uptake of drugs and nanoparticles and their intracellular delivery by molecular motors for therapeutic processes [23].

**Figure 1:**
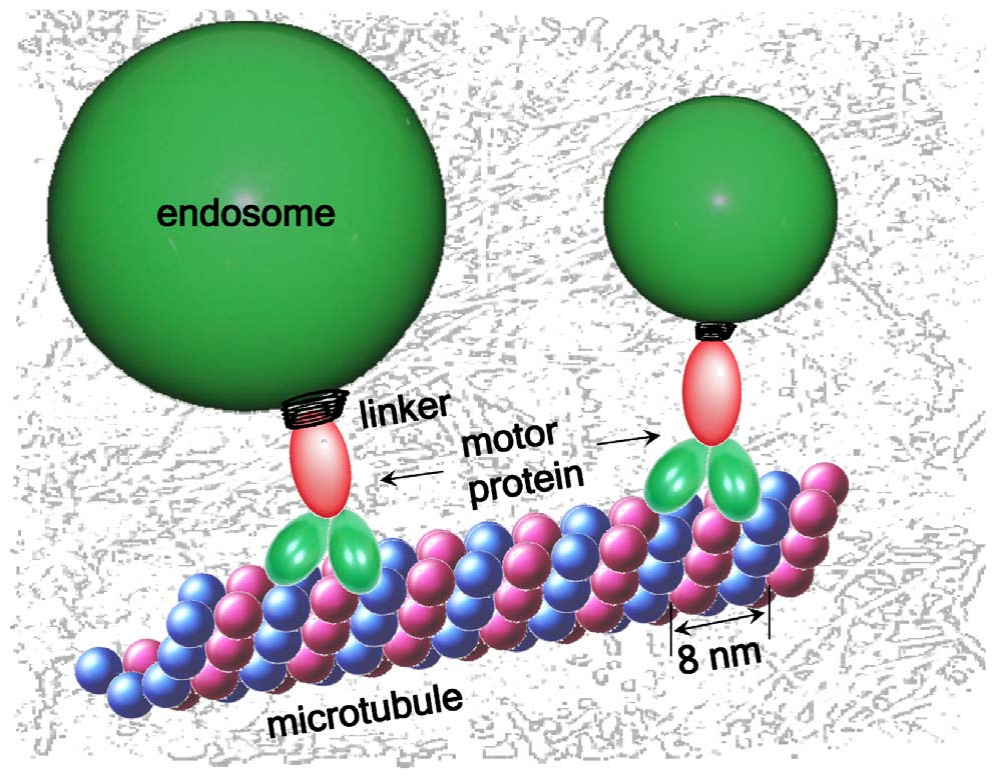
Molecular motors moving along a microtubule in the crowded cytoplasm. A large cargo is subject to viscoelastic drag, effecting dramatic changes in the transport dynamics.

What happens to the active motion of particles in the cytosol which are driven by molecular motors [24]–[32], see Figure 1? Will the overall dynamics of the coupled motor-cargo system be affected by the superdense state of the cytosol, and how? In fact, anomalously fast diffusion was observed in living cells for the active motion of viruses, microbeads, and endosomes [16], [17], [33]. When the motors normally transport their cargo with some mean velocity in a given direction, ballistic superdiffusion with 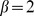 is measured [16]. However, various subballistic power exponents 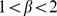 are also found [17], [33]. Here we come up with a mesoscopic physical approach for molecular motors in the cytosol of living cells and show that our predictions are in agreement with the experimentally observed behavior. The simplest explanation for the experimental fact that transport by motors along intracellular microtubuli yields subballistic superdiffusion (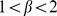), is provided by the sublinear scaling 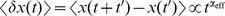 of the mean position 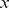 along a single microtubule, with an effective scaling exponent 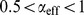. Then, assuming quenched disorder in microtubuli orientations without a preferred direction yields 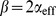 which will range in the subballistic superdiffusive regime. Can normal and anomalous transport with various 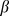 co-exist and be mediated by the *same* motors in the *same* cell? Which role is played by the size of the cargo, and what determines the precise behavior of such active transport and its efficiency? These are the questions we answer in this article.

A well-established physical approach to anomalous transport phenomena is based on the intrinsic viscoelasticity [5], [34]–[37] of complex fluids such as the cytosol. Depending on its size and speed, a nanoparticle may feel different effective viscosities in the macroscopic limit of normal diffusion which may be enhanced by a factor of hundred to several thousands with respect to the viscosity of pure water [38]–[40]. Transient regimes of anomalous diffusion become biologically important when the corresponding spatial length scale is comparable to the linear size of cells, typically several micrometers. The viscoelastic nature of the cytosol and other dense solutions has been verified in several experiments [19], [20], [41], [42].

## Results

### Ratchet Model of Molecular Motors

**Figure 2:**
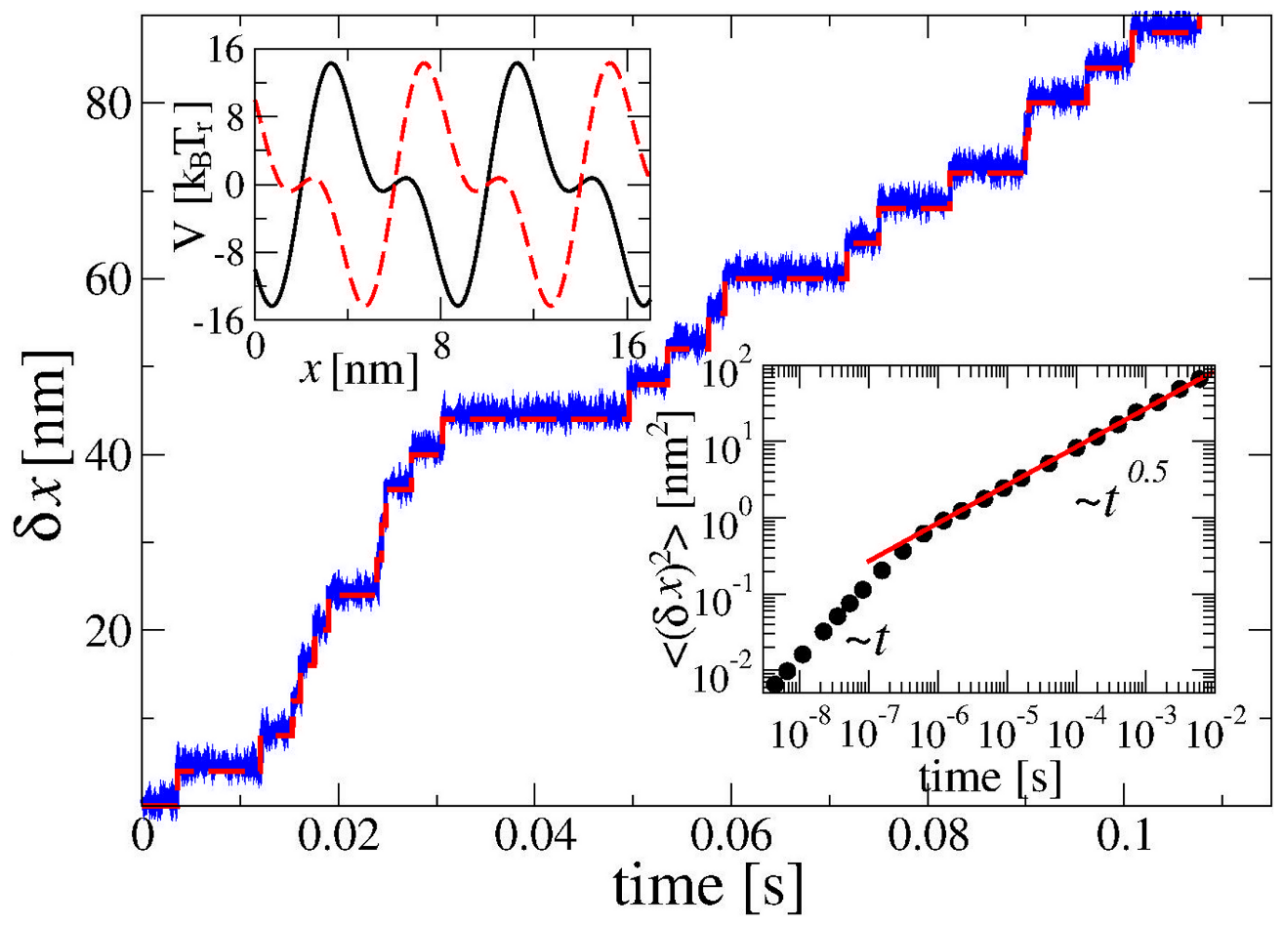
Normal transport for large cargo particles, large potential amplitude and small turnover rate, in the absence a of constant loading force, 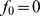. Single motor transport (full line) is almost perfectly locked to the potential fluctuations (broken red line depicting a renewal process counting the number of potential fluctuations in units of 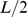) occurring with mean turnover frequency 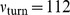 Hz, in a potential (top inset) with amplitudes 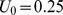 eV (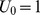 in dimensionless units) and 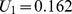 eV (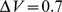 eV), for 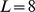 nm. A particle with an effective radius 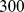 nm (like a magnetic endosome [33]) experiences asymptotically for 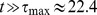 sec an effective viscous friction enhanced by a factor of 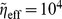 with respect to water. The bottom inset shows that on the relevant transient time scale the free particle subdiffuses with anomalous diffusion coefficient 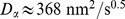. Initially, diffusion is normal. The time-average over a single trajectory, 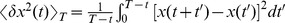, is shown for 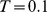 sec and compared with the theoretical subdiffusive ensemble-averaged result (red line). See **Methods**.

We study the interplay of the viscoelastic environment of the cytosol with the action of a molecular motor and its cargo. A well-established model of Brownian motors of the kinesin family is based on the continuous diffusion of a Brownian particle in a potential landscape, which randomly fluctuates in time between two realizations, 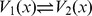, depending on the internal state of the motor, that is undergoing active conformational fluctuations [25]–[29], [31], [32]. These conformational fluctuations are caused by the binding of negatively charged ATP molecules to the motor (state 1) and the reactions of ATP hydrolysis and dissociation of products (ADP and phosphate group) making up state 2 within a minimalist modeling framework. The potentials 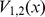 describe the free energy profiles leading the motor molecule in the corresponding conformational states along a microtubule. Since microtubules are periodic dipolar structures with period 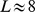 nm, the potentials reflect this periodicity, 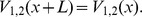 Moreover, 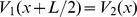 within our two-state motor model, such that two potential switches occur during one cyclic turnover of the motor enzyme (power-stroke or “hand-over-hand” mechanism) and advance the motor by one period 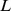. The motor direction is determined by the polarity of the microtubules, reflected in the space inversion asymmetry of the potentials. We use the harmonic mixing model [32] with 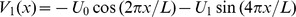, see the upper inset in Figure 2. This potential has a metastable state within each period. Thermal fluctuations play a positive role, allowing the motor to avoid getting trapped in such metastable states on its search for the potential minimum after each conformational change due to ATP binding and ATP hydrolysis.

Within the power-stroke idealization, the maximal mean velocity of the motor due to the fluctuations between the potentials 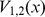 becomes 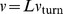, where 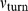 is the typical motor turnover rate. The latter is composed of the conformational transition rates 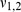 according to 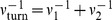. As the simplest approach based on the widely applied flashing ratchet model [26], [29], [31], [32], [43], we assume that these transition rates do not depend on the transport coordinate 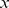. Such an ideal motor would consume one ATP molecule (with an energy amount of 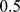 to 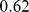 eV or 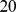 to 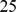 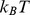 at room temperature, 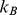 being the Boltzmann constant) while transferring a cargo over the distance 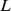. The corresponding energy 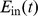 invested into the temporal increase of the potential energy of the motor during repeating turnover cycles of the ‘catalytic wheel’ [44] can be calculated as a sum of potential energy jumps 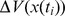 occurring at random instants of time 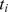 marking cyclic conformational transitions 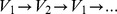 [45]. Furthermore, apart from delivering a cargo over a distance 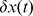, the motor can perform useful work, 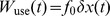, against some constant force 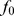 opposing its direction of motion. Ensemble averaging over many trajectory realizations, we obtain the thermodynamic efficiency 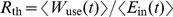. In the long time limit this is a time-independent quantity in the normal transport regime, where both 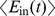 and 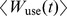 are proportional to time. If 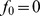, the thermodynamic efficiency is zero, and all the input energy will be dissipated as heat. In order to characterize the energetic performance of a molecular motor in such a situation, we introduce the delivery efficiency 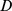 defined as the ratio of delivery distance 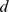 to the product of delivery time 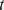 and the average number of turnover cycles 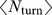 (number of ATP molecules consumed). 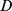 thus has the meaning of a mean delivery velocity per input energy amount (in dimensionless units). The goal is to deliver cargo over a certain distance as quickly as possible using the smallest amount of energy. Thus, ideally 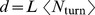 and 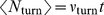. Therefore, 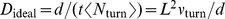, which linearly increases with the turnover frequency at some fixed 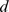. However, we expect the delivery efficiency to deviate from this idealization.

Both the motor and its cargo are subjected to friction and random thermal forces from the environment. For normal viscous Stokes friction, the frictional drag force is 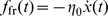, where 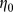 is the friction coefficient. It is proportional to the medium’s viscosity 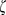 and the particle size. For a sphere of radius 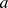, 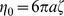. If we assume that the linker between the motor and its cargo is rigid, we can model their coupled motion as that of a point particle moving under an effective frictional force. In this way, one accounts for the cargo size simply by adjusting the effective friction. The dynamics for such a simplified motor is then defined by the Langevin equation
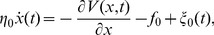
(1)where 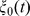 is a Gaussian random thermal force with zero mean, which is completely characterized by its autocorrelation function. The fluctuation-dissipation relation 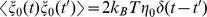 ensures that the description is compliant with the laws of thermodynamics, so that no directional motion can emerge when 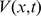 is time-independent and fixed to either 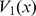 or 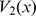. The prototype motor model (1) has been investigated in great detail within a Markovian setting, using various model potentials and a different number of motor substates [25]–[27], [29], [32], [43].

### Viscoelastic Environment

The above ratchet model is appropriate to describe the motor dynamics at dilute solvent conditions in vitro. Our focus here is different, as we want to study the motor action in living cells, where the following experimental facts have been established: Even in the absence of cargo the effective friction coefficient for the motor is enhanced by a factor of 100 to 1000 in the cytosol compared with the one in water [28], [38]. This phenomenon is due to the superdense state of the cytosol, crowded with various biopolymers. Concurrently, the diffusing nanoparticles themselves experience a medium with an effectively enhanced viscosity, that can exponentially depend on the particle size [39], [40]. Moreover, numerous experiments reveal [20], [33], [36], [37] that the complex shear modulus 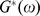 of the cytosol displays a power law scaling 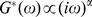, 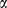 ranging between 0.2 and 0.9 for frequencies in the range from inverse milliseconds to several hundred inverse seconds [5], [9], [20], [33], [37]. This reflects the viscoelastic nature of the cytosol, which needs to be taken into account for molecular motors [17], [33], [34], [46]–[51]. To explicitly consider viscoelastic effects for cargo particles is even more pressing given the experimental results revealing viscoelasticity-induced subdiffusion of free, passively moving submicron particles [17], [20], [21], [41], [42], whose size is comparable to typical, larger cargo such as vesicles. This implies that: (i) the dynamics must be described by a frequency-dependent friction corresponding to a viscoelastic memory for the friction term with a power-law kernel [34] 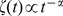, such that (ii) the diffusion of free particles becomes anomalously slow 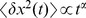 with 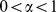 on the corresponding time scales from milliseconds to minutes. This is a mesoscopic, transient effect. However, it becomes very important for transport processes in living cells as they occur on exactly the physiologically relevant time and length scales. Consequently, the Langevin equation (1) for the motor’s velocity must be extended to the generalized Kubo-Langevin form
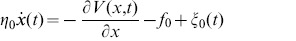





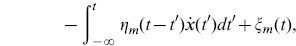
(2)where 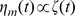, and 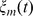 represents colored thermal Gaussian noise with autocorrelation function 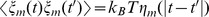, as demanded by thermodynamics (absence of directed transport in a static periodic potential 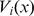) and the Kubo second fluctuation-dissipation theorem [52]. On physical grounds, a memory cutoff always exists for 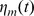 such that on a sufficiently long intermediate time scale the kernel has the scaling property 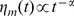. This ensures that the effective friction coefficient 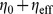 with 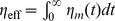 is finite, but strongly enhanced over 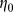, in compliance with previous studies of molecular motors [28]. There exists also a short time cutoff, corresponding to the largest vibrational frequency of the medium contributing to the friction. Given the upper and lower cutoff, one can approximate the memory kernel 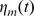 by a finite sum of exponentials [34], [53] and the generalized Langevin equation (2) can be derived from a corresponding multi-dimensional Markovian Maxwell-Langevin model of viscoelastic dynamics [34], see **Methods** for details. On the basis of this theoretically and experimentally well-founded approach the results herein were obtained from numerical analysis.

### Perfectly Normal Transport

The first major surprise is that even carrying large cargo particles like magnetic endosomes with radius about 300 nm our model motor can operate by an almost perfect power stroke mechanism in the normal transport regime, as demonstrated in Figure 2. For this, the binding potential amplitude should be sufficiently large, 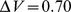 eV, and the turnover rate sufficiently small, 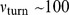 Hz, both reasonable values for this motor-cargo system. This almost perfect dynamics occurs in spite of the fact that the free cargo alone subdiffuses, 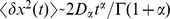, on a transient time scale between 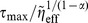 (see **Methods**) and 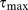 with a subdiffusion coefficient as small as 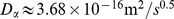 for 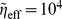, 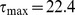 sec, and 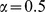, an experimentally relevant value we consider in this work. This normal transport regime is possible as viscoelastic subdiffusion is ergodic [8], [34], [53]. Moreover, for a sufficiently large potential barrier 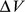 separating spatial periods of the potential the particle has enough time 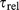 to relax and settle down sufficiently close to the potential minimum. Thus, it can be advanced by a half-period at each switch of the potential, despite the power-law character of the relaxation dynamics, if only the time scale separation condition 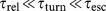 is satisfied. This condition is easy to fulfill for realistically small turnover frequencies 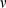 and realistic 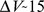 to 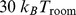 since the mean escape time grows exponentially fast with the barrier height, 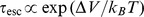. The occurrence of such normal transport is consistent with most observations on molecular motors. Thus we accomplished our first goal, to explain the active normal transport of particles, that otherwise subdiffuse when they are not attached to a motor. However, the model motor considered here is actually assumed to be a little too strong. It has a stalling force of about 15 pN, almost twice the typical value, see below. Let us therefore consider a somewhat weaker binding potential and faster enzyme turnover rates to see the origin of anomalous transport for such a large cargo.

### Anomalous Transport

**Figure 3:**
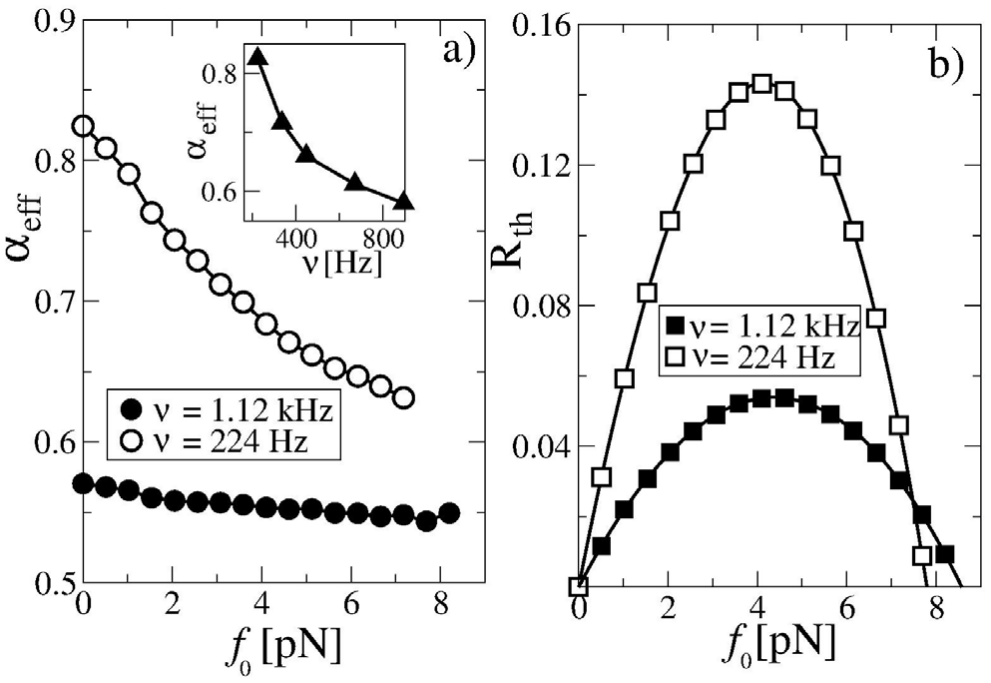
Anomalous transport of large cargo particles at lower potential amplitude, larger turnover rates, and in the presence of loading force 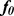. (a) Effective anomalous transport exponent 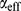 and (b) thermodynamic efficiency 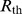 while working against a constant force 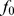 near the end point of the simulations (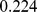 sec or 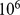 in dimensionless units). The thermodynamic efficiency decays over time as 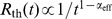. The analysis considers the same particles as in Figure 2, but here the potential height is reduced by factor of 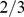. Ensemble averaging is performed over 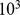 particles and random realizations of potential flashes. The inset in (a) shows the dependence of 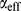 on the mean enzyme turnover frequency for 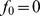.

Indeed, when we decrease the potential amplitude by 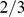, and consider an increasing turnover frequency in the presence of an opposing (external drag) force 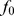, which further reduces the effective barrier height, an anomalous transport regime 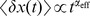 with 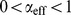 is enforced, see Figure 3,a. The thermodynamic efficiency in this strongly anomalous transport regime is rather small, see Figure 3,b. Moreover, it becomes an algebraically decaying function of time [50], [51], 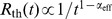. This happens because 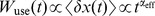 scales sublinearly with time, while the input energy is consumed at a constant rate. This means that asymptotically most of the input energy is used to overcome the dissipative influence of the environment characterized by the massively enhanced effective viscosity. Concurrently, the useful work performed against the force 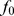 always remains finite and the stalling force 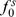 is also about the same as for normal diffusion ratchets. This finding agrees with typical experimental values of 7 to 8 pN for the stalling force of kinesin motors [28]. The slower the motor turnover is, the larger the effective transport exponent 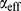 becomes, along with a higher motor efficiency. The efficiency displays almost a parabolic dependence on the force 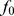, 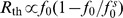 in this regime. A similar dependence was derived for fluctuating tilt viscoelastic ratchets [50], [51]. The stalling force is not only roughly proportional to the barrier height, but also depends on the frequency of the potential switches.

### Smaller Cargo Size

**Figure 4:**
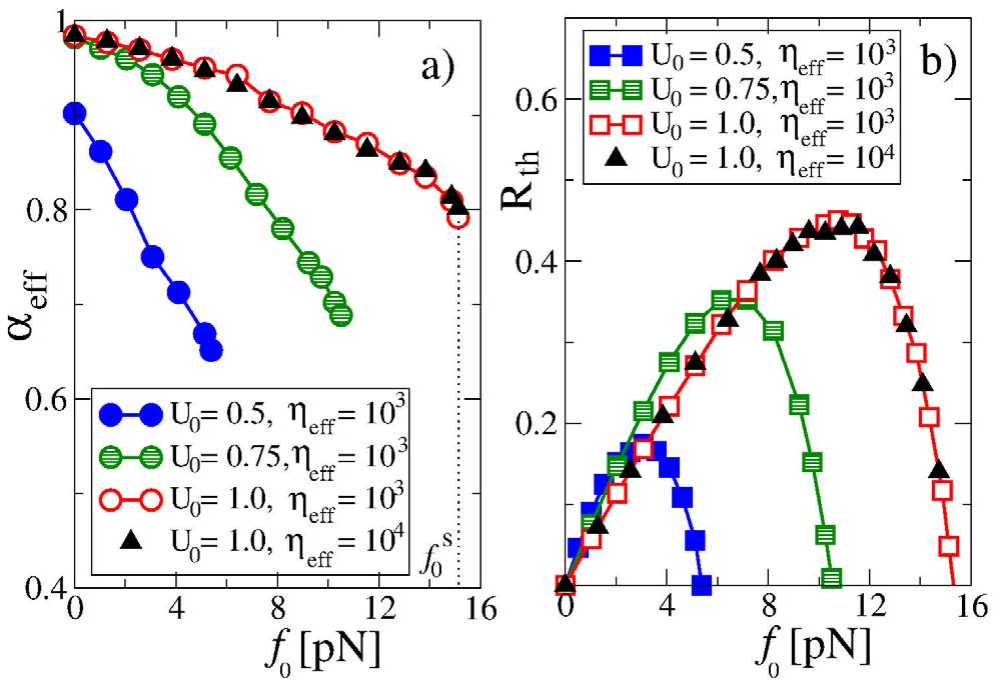
Dependence of (a) the effective transport exponent 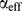 and (b) the thermodynamic efficiency 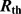 on the load 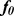, for three different potential amplitudes and for turnover frequency 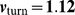 kHz. Ensemble averaging is done over 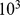 particles and random realizations of potential flashes, 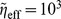, 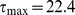 sec, or 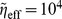 and 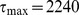 sec (with the same 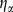, see **Methods**). Matching of the results for two sets with the same amplitude 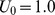 indicates that 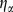 is the characteristic quantity, rather than 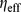 and 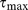 separately. Efficiency is calculated at the end point of simulations 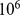 corresponding to 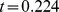 sec.

Depending on the cargo size and the binding potential amplitude the transport can become more normal and thermodynamically highly efficient even for a large turnover frequency, as Figure 4 illustrates for 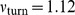 kHz. This is about the maximal turnover frequency which can be expected for molecular motors. For this, the potential amplitude should be sufficiently large and the cargo smaller in size. Here, we reduced 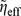 to 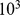 with the same 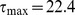 sec. Hence, assuming that the effective viscosity of the medium remains unchanged, 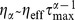 becomes reduced by a factor of ten, which corresponds to a cargo with one tenth of the size, that is, of some 30 nm radius. However, if we were taking into account its dependence on the particle size [38]–[40], this value of 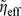 should in fact be attributed to a somewhat larger particle. The subdiffusion coefficient is enhanced accordingly, 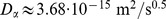. Furthermore, to show that the fractional friction strength 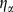 and the subdiffusion coefficient 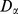 are characteristic for the transport properties rather than 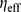 and 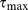 separately, we also considered the case with 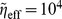 and 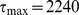 sec yielding the same 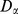, see data with 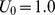 in Figure 4. For the largest potential amplitude in Figure 4 the thermodynamic efficiency is appreciably high, up to 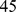
% for the studied case. This is very surprising: The transport efficiency in the anomalous regime can be temporally almost as high as the maximal efficiency of kinesin motors in the normal regime (about 50%). For this potential amplitude, however, our motor is stronger than a typical kinesin motor. It has a stalling force of about 15 pN, see Figure 4. The effective exponent 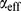 is about 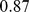 at the maximum corresponding to 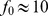 pN. However, the transport is anomalous and the efficiency decays algebraically as 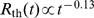. For increasing loading force 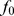 the anomalous diffusion exponent 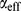 becomes smaller and the thermodynamic efficiency drops faster as function of time. This means that the optimal value of the force 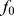 corresponding to the maximum of 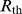 slowly shifts towards smaller values, as if the motor became gradually ever more tired, and more quickly exhausted for a higher load. Upon reduction of the barrier height by a factor of 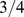 the transport is still close to normal for 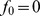. Moreover, the thermodynamic efficiency can still be temporally rather high at optimal load. However, 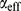 now drops faster with 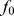. A stronger reduction of the potential amplitude, by one half, leads immediately to the emergence of a low efficiency, strongly anomalous transport regime, even for 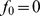 (Figure 4). Still, a strong reduction of the turnover frequency down to 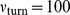 Hz will recover the normal transport regime for a sufficiently small 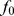 in all cases considered. The transport of even smaller particles is clearly normal for realistic parameters.

### Delivery Performance

**Figure 5:**
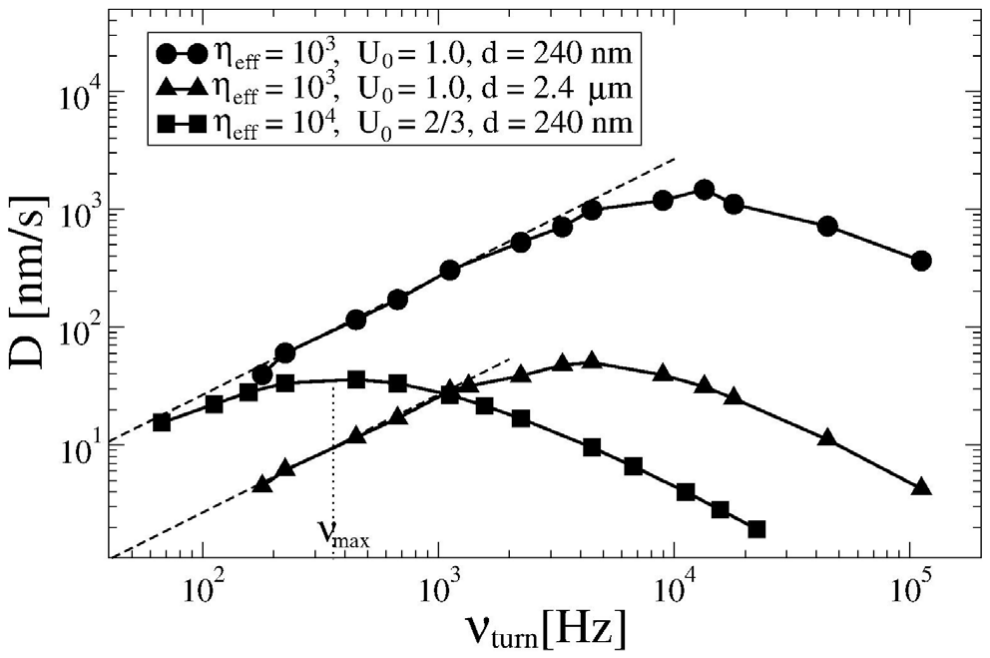
Delivery efficiency 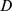 as function of the turnover frequency 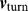. In the ideal power-stroke regime, 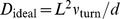. For small 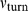 our results agree with this simple dependence (broken lines).

For vanishing loading force 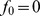 thermodynamic efficiency is zero, even in the normal transport regime. We proposed above that the performance of molecular motors such as kinesin should be characterized by the energetic efficiency 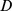 of the cargo delivery over a certain distance 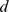. We calculated the above-defined delivery efficiency 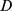 in Figure 5 as function of the turnover frequency 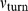 for several different delivery distances 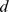, potential amplitudes 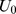, and fractional friction coefficients 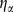. Remarkably, for a small cargo (smaller 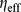 in Figure 5) the calculated delivery efficiency follows the ideal power-stroke dependence, 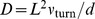 in the entire range of realistic turnover frequencies. Even for a relatively large cargo, Figure 3, but for much smaller turnover frequencies the transport is close to this ideal normal regime. It is expected to be normal already for 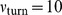 Hz. Such a small frequency is, however, not accessible to numerical analysis. In the anomalous regime, with increasing turnover frequency the delivery efficiency reaches a maximum at 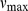 and then decreases. The anomalous transport becomes less efficient for 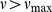 and 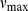 shifts to smaller values with increasing delivery distance 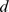. We speculate that in the cell economy this effect can become relevant for the optimization of the metabolic budget.

## Discussion

We proposed a simple basic model which reconciles experimental observations of both normal and anomalous transport by highly processive molecular motors in biological cells. Our model presents an immediate generalization of a well-known two-state model of normally diffusing molecular motors by accounting for the viscoelastic properties of the intracellular fluid. It not only explains how molecular motors may still operate by a power-stroke like mechanism while carrying a large cargo which subdiffuses when left alone, but also why and how an anomalous transport regime emerges for even larger cargo. It is crucial for this explanation that viscoelastic subdiffusion and anomalous transport possess finite moments of residence times in any finite spatial domain. Thus, there exist time scales for sliding down towards the potential minimum within one period of the flashing ratchet potential, for the escape to another potential well, and for the mean turnover time of the potential flashes. When the time scales are well separated, the transport is normal. However, when the sliding time scale starts to interfere with the turnover time, or the interwell potential barrier is lowered so that backsteps can occur, the transport becomes anomalous. These qualitative basic features are expected to survive in more complex models of molecular motors operating in viscoelastic environments.

Specifically, we showed that transport by molecular motors becomes anomalous for large cargo particles with large fractional friction coefficient 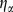 when the enzyme turnover is fast, and the binding potential amplitude 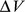 is not sufficiently large. Larger potential amplitude 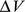 for a fixed spatial period leads to faster relaxation of the motor particle to a new potential minimum after each potential switch and thus to more normal transport behavior. The enhancement of 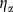 in contrast leads to slower relaxation which asymptotically follows a power law decay 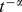. For this reason, to secure the occurrence of normal transport the condition 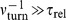 must be well assured, with time scales separated by several orders of magnitude. Since the potential curvature can be estimated as 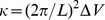, and the anomalous relaxation rate as [53] 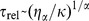, one can see that the ratio 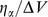 is important to determine the scale of 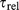. Even if the transport of a large cargo is anomalous for 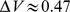 eV in Figure 3, the reduction of 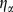 rapidly enforces normal transport, similarly to the reduction of 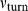. Therefore, for realistic turnover frequencies the molecular transport by kinesins is expected to be normal for vesicles of a typical radius of 30 nm, and possibly up to 100 nm. Anomalous transport emerges for above cargos with radius 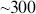 nm or larger. This explains why the same molecular motors can mediate both normal and anomalous transport in living cells depending on the cargo size. The occurrence of an anomalous transport exponent is thus reconciled with the normal transport behavior for small cargo at lower turnover frequencies.

Our research provokes a number of follow-up questions. Thus, what happens if we relaxed the assumption of a rigid motor-cargo linker molecule? In that case, the large subdiffusing cargo is elastically coupled to a molecular motor, that possibly still operates normally in the absence of cargo. We are currently investigating this generalization for realistic spring constants of the linker. However, qualitatively the results remain very similar. Another question is prompted by the experimental results in Ref. [33], suggesting that the motors can collectively transport several magnetosomes jointed into a chain. A generalization of our non-Markovian model to such collectively operating motors would be important for our understanding of large-cargo transport in living cells.

Our model presents a good starting point for future research and further generalizations. Understanding how molecular motors perform in the viscoelastic cytosol of living cells despite the subdiffusion of the free cargo is compelling. Our findings open new vistas to the old problem of intracellular trafficking, reconciling seemingly conflicting results for the motor-cargo dynamics under different conditions. Finally, our results will be of crucial importance for the design of new technologies of motor-driven particles and drug delivery in the crowded cytosol of cells. We are confident that our findings will prompt a series of new experiments on the dynamics of molecular motors under realistic conditions in living cells.

## Methods

The numerical approach to integrate the generalized Langevin equation (2) with a power-law memory kernel rests on the approximation of the memory kernel by a sum of exponentials [34], [53],
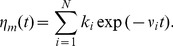
(3)


The rate constants 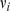 and elastic constants 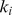 are chosen to obey a fractal scaling [54], 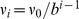, 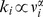, with a dilation parameter 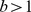, and a shortest memory time 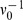 in the hierarchy. Due to the scaling property 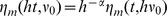 this choice indeed provides a power law regime, 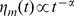 on time scales 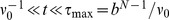, with small superimposed logarithmic oscillations. Physically, this corresponds to representing a viscoelastic environment by auxiliary Brownian quasi-particles with coordinates 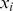. They are coupled to the central Brownian particle by elastic constants 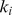 and subjected to the thermal noises and frictional forces with viscous frictional constants 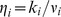 [34],
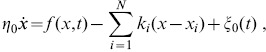





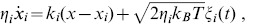
(4)where 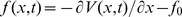, and 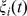 are uncorrelated white Gaussian noises of unit intensity, 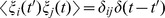, which are also uncorrelated to 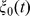. To have a complete equivalence with equations (2), (3), the initial positions 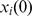 are sampled from a Gaussian distribution centered around 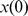, 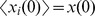 with variance 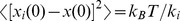. We set
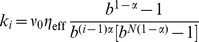
(5)and use 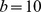 which leads to a maximal relative error with respect to the exact power-law of less than 4% [50], for 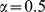, on relevant intermediate time scale. The effective relative friction coefficient 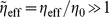 is used as a parameter in our simulations. The parameter 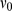 controls the matching accuracy of our model with the model of fractional Langevin dynamics (the memory kernel 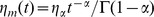) at short to intermediate times, where initially for times 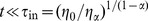 [51] the free diffusion is normal, 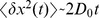 with 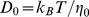 in accordance with the Einstein-Stokes relation. At longer times, anomalous diffusion emerges, 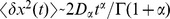 with 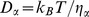. The number 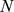 of auxiliary quasi-particles controls the maximal range of subdiffusive dynamics, after which diffusion becomes again normal, 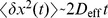, for 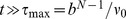 with 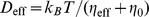. The fractional friction coefficient is 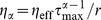, where 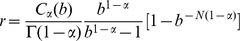 is a numerical coefficient of the order of unity, 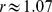 for 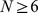, 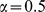, and 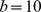, with 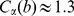 [53]. An interesting observation is that in terms of 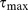 and 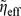, 
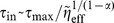
. Therefore, the effective viscosity 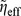 defines the time range of subdiffusion, from 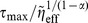 to 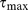, independently of 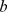, and 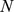
! For example, for 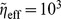 and 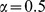 one expects that subdiffusion will extend over 6 decades in time. In the simulations, we scale length in units of 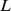 and time in units of 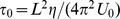, where 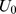 was taken equal to 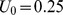 eV (or about 10 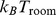 with fixed temperature). The time step was 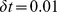 and 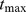 was varied from 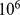 to 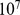. The ratio 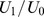 was fixed to 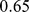. Four different values of 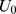 were used: 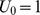, 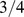, 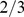, and 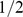. The largest one corresponds to the largest potential barrier 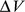 separating two potential periods of about 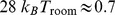 eV, 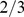 to about 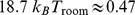 eV, and the smallest one to 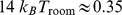 eV. The larger 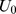 the more efficiently the motor works, as the probability of thermally activated backsteps is exponentially suppressed with 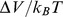. This is necessary to provide an ideal power-stroke operation at small turnover frequencies. However, the energy released from the hydrolysis of one ATP molecule will not be sufficient to perform one cycle of operation in the case 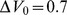 eV because of the energy derived from the hydrolysis of one ATP molecule does not exceed 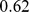 eV. In such a case, to drive a cycle of two potential flashes one needs an input energy of at least 0.7 eV (0.35 eV per one conformational change, about one half of potential barrier separating potential periods, see upper inset in Figure 2).

The power exponent of anomalous diffusion was fixed to 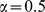 to interpolate between 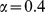 for an intact cytoskeleton [33], [46] and 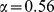 [33] when the actin filaments are disrupted. For the radius 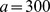 nm of a single magnetic endosome [33] one estimates 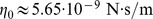 in water of viscosity 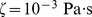. This yields 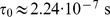. Furthermore, we use 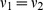 in the simulations. Hence, 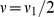, and the frequency 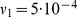 corresponds to 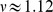 kHz. This is about the maximal possible turnover frequency for molecular motors [28]. For an effective particle ten times smaller, 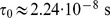 and 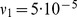 would correspond to the same physical turnover frequency. For a magnetic endosome we estimate 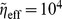, 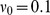 and take 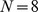, which yields 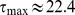 sec and 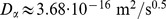, of the same order of magnitude as in experiments for a cargo consisting of several such endosomes [33]. Alternatively, with a smaller 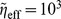 for 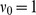 and 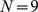, 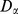 is by a factor of ten larger, 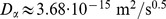, at the same 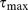. One obtains the same 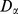 by simultaneously increasing 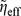 to 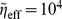 and 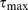 to 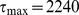 sec (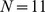).
